# United States Under-19 Rugby-7s: Incidence and Nature of Match Injuries During a 5-year Epidemiological Study

**DOI:** 10.1186/s40798-020-00261-y

**Published:** 2020-08-27

**Authors:** Victor Lopez, Richard Ma, Meryle G. Weinstein, Patria A. Hume, Robert C. Cantu, Christian Victoria, Sophie C. Queler, Khalil J. A. Webb, Answorth A. Allen

**Affiliations:** 1 Rugby Research and Injury Prevention Group, affiliate Hospital for Special Surgery, 118-17 Union Turnpike, Suite 3B, New York, NY 11375 USA; 2 Auckland University of Technology, Sports Performance Research Institute New Zealand, New Zealand, AUT Millennium, 17 Antares Place, Mairangi Bay, Private Bag 92006, Auckland, 1142 New Zealand; 3 USA Rugby Empire Geographic Union RFUs, P.O. Box 488, Bowling Green Station, New York, NY 10274 USA; 4 USA Rugby New England Geographic Union RFU, 2193 Commonwealth Ave, Box 364, Brighton, MA 02135 USA; 5 Northeast Rugby Academy, USA Rugby Development Program and USOC-Community Olympic Development Program, New York, NY USA; 6 University of Missouri, Missouri Orthopaedic Institute & Thompson Laboratory for Regenerative Orthopaedics, Columbia, MO USA; 7 New York University, Department of Applied Statistics, Social Sciences, and Humanities, New York, NY USA; 8 Center for the Study of Traumatic Encephalopathy, Boston University School of Medicine, Boston, MA USA; 9 Cantu Concussion Center, Department of Neurosurgery and Sports Medicine, Emerson Hospital, Concord, MA USA; 10 Neurologic Sports Injury Center, Brigham and Women’s Hospital, Boston, MA USA; 11 Concussion Legacy Foundation, Waltham, MA USA; 12 World Rugby, Independent Concussion Group, Dublin, Ireland; 13 New York University, College of Global Public Health, New York, NY USA; 14 Tulane University, New Orleans, LA USA; 15 University of Arizona, Tucson, AZ USA; 16 Sports Medicine Institute, Hospital for Special Surgery, New York, NY USA; 17 National Basketball Association, New York Knickerbockers, New York, NY USA; 18 USA Basketball, Colorado Springs, CO USA

**Keywords:** Adolescent, Rugby union sevens, Community, Sports injuries, Epidemiology, Risk factors, Concussion

## Abstract

**Background:**

There is a lack of injury data for the new Olympic sport of Rugby-7s, particularly for involved youth.

**Objective:**

To determine injury rates and characteristics for players participating in U.S. Rugby-7s U19 (under 19 years of age) tournaments.

**Methods:**

Injury data were collected, using the Rugby Injury Survey & Evaluation report methodology, at 24 U.S. Rugby-7 s U19 tournaments over 30 tournament days (2010–2014). Tournament medical-attention injuries and time-loss injuries (days absent before return to training/competition including post tournament) were recorded.

**Results:**

During the 2101 playing hours (3072 males, aged 17.2 ± 1.5 years; 732 females, 16.6 ± 1.3 years of age), there were 173 tournament injuries with an overall injury incidence of 82.4/1000 player-match-hours (ph) (CI 70.5–95.6). Acute injuries (79.5/1000 ph) occurred during tackling (56.2/1000 ph) and involved joints/ligaments (32.8/1000 ph) of lower extremities (31.9/1000 ph). Head and neck injuries, including concussions, were common (males 21.9/1000 ph; females 22.0/1000 ph). Medical-attention injury incidences (49.5/1000 ph; *n* = 104; 95% CI 40.5–60.0) were higher than time loss (32.8/1000 ph; *n* = 69; 95% CI 25.5–41.6). Overall, injury incidences found no difference between sex (RR 0.78; *p =* 0.369). Time-loss injuries resulted in an average of 35.5 d to return to sport.

**Discussion:**

This study is the first to report match injury incidences for U19 participants in Rugby-7s. Overall, match injury incidence among U.S. U19 Rugby-7s tournaments was similar compared to adult U.S. community Rugby-7s. Recurrent injury risk was notable in this population. Community injury surveillance studies are essential to understand risk from participation in amateur sports. Knowledge of these injury patterns in U19 Rugby-7s will help identify areas to direct resources to enable growth of Rugby-7s in youths and emerging countries being exposed to Rugby-7 s. Age-based injury frequency and patterns in rugby and its various formats are needed for the development of evidence-based, sport-specific, and population-specific injury prevention initiatives.

**Conclusions:**

The match injury incidence of U19 participants in U.S. Rugby-7s was similar to the incidence among adult participants. Recurrent match injury risk was high at 23%. There were no significant differences in injury incidences between males and females. The first three matches of a tournament day result in the most injuries.

## Key points

- Overall, injury incidence for Rugby-7s U19 players was 82.4/1000 ph (including 69 time loss, 32.8/1000 ph; and 104 medical attention, 49.5/1000 ph; *p =* 0.014).
- Adolescent players sustained 90% of all their injuries and 88% of all time loss greater than 28 days absence during the first three matches of a tournament day.
- Most injuries were contact (64.3/1000 ph) compared to non-contact injuries (6.7/1000 ph; *p <* 0.001). Most injuries were a result of impact with another player (60%) as opposed to impact with another entity (i.e., ground or ground and player) (40%; RR 1.5: *p* = 0.020).
- Recurrent match injury risk in the U19 Rugby-7 population was 23%.
- The most common time-loss injuries were concussions (6.2/1000 ph; 34d), ankle ligament injuries (5.7/1000 ph; 16d), and shoulder ligament injuries (2.4/1000 ph; 23d).
- There were no significant differences in injury incidences between males and females.

## Background

Rugby union, which will be referred to as “rugby” from this point forward, has gained recent popularity globally and within the United States of America (U.S.) [1]. The two popular formats of Rugby union in the USA are Rugby-15s and Rugby-7s, which are played by both males and females. Rugby-7s differs from Rugby-15s in the number of players on the field (7 vs 15), match length (7- vs 40-min halves), and match frequency (3–6 matches per day in 7s vs 1–2 matches per week in 15s) [2]. The sport’s growth in the USA is evident by the U.S. (> 1.5 million participants) trailing only England in estimated total number of participants based on World Rugby data [3, 4].

Approximately, half the world’s 7.23 million rugby players are under 19 years old [3]. Levels of youth play according to World Rugby comprise the pre-teen age groups and vary from introductory at Under-6 to Under-8, Under-10, and Under-12. Meanwhile, adolescents and teenagers can be categorized by ages as pre-teens (Under-12), teens (Under-14 and Under-16), and senior-age players (Under-19 and up) [5]. The USA has seen exponential growth in rugby participation among its youth and adolescents and is considered a strategic market [1].

There are limited Rugby-7s injury data [6–10], including on U.S. players [2, 11–13]. This is partly due to rugby having only club status in the U.S. sporting landscape, which results in its exclusion from existing injury surveillance systems [14–16]. Internationally, youth and adolescent rugby injury literature are exclusively on Rugby-15s, which utilize differing methodologies and definitions, resulting in varying injury incidences (1.6–129.8/1000 ph) [17–20]. As Rugby-7s is a more open format having less players on the same size field as Rugby-15s, it is played differently and requires different physical demands compared to Rugby-15. As a result, injury patterns between the two formats are different with Rugby-7s typically having higher incidence of injury compared to 15s [6, 21].

In addition to a comparative lack of data on Rugby-7s, there is also no information currently on injury risks with youth participation in Rugby-7s. Compared to adults, youths may be more at risk of injury due to comparatively limited exposure to Rugby-7s and learning the sport skills, and also due to their changing physical and psychological growth and development [5]. A disparity in size and development of motor skills may result in competitive disadvantages and in the rise of injuries in these age groups [5]. There is, therefore, a need for injury data in youth participation in Rugby-7s.

The aim of this study was to determine U.S. Rugby-7s U19 injury incidence in matches according to international standards on injury research in rugby [22]. Our hypothesis was that incidence and nature of injuries among U.S. Rugby-7 s U19 players would be different compared to current literature on adult Rugby-7s. Data from this study will guide population-specific injury prevention among youths participating in Rugby-7s.

## Methods

### Study Design, Ethics, and Data Collection

Ethics approval was obtained from the Hospital for Special Surgery (IRB #11025, #2014-205CR1), and the study was performed in accordance with the standards of ethics outlined in the Declaration of Helsinki. A prospective observational cohort design was used. Injured U19 Rugby-7s athletes participating in U.S. Rugby-7 s tournaments between 2010 and 2014 were recruited into the study. Injured athletes older than 18 provided informed written consent for their injury data to be used in the study. Written consent from parents/guardians and assent were required from injured athletes younger than 18 years to participate. Research coordinators/research data collectors (RC/RDC) (minimally, a study-trained healthcare provider, athletic training student, or certified athletic trainer capable of clinical decision-making) documented data for match injury. Match injuries were identified by players that were brought to the tournament-sanctioned medical tents or injuries that were seen to occur on the playing pitch by the sideline tournament-sanctioned healthcare providers. To standardize the study across multiple events, a RISE (Rugby Injury Survey and Evaluation) study research information package was sent to tournament directors and disseminated to all teams in advance of each tournament. The package included IRB approved details of the study protocol, including all study sanctioned assents/consents for players. The package included descriptions of the RISE report and the proper use in cataloging for the RC/RDC. Procedures for the team’s study-approved RC/RDC and follow-up of injured players following the tournament in order to obtain the return-to-sport time window were also described. Tournament schedules were obtained which provided an exactness to calculating playing exposure [2, 11–13].

### Participants and Events

Participants from 24 U.S.-based Rugby tournaments (3 in 2010; 4 in 2011; 5 in 2012; 2 in 2013; 10 in 2014), spanning 30 tournament days (25 natural grass, 5 artificial turf), were included. Events included only tournaments played within the USA: in the USA Rugby (USAR) Geographic Union (GU) 7s Series, the USAR Territorial Union/Competitive Region (TU/CR) Qualifier Championship Series (in 8 U.S. states/regions of the USA), and a Sevens Invitational in the southwest of the USA. Each team played minimally 3–5 matches per event, and two teams with the best performances played an additional final round match (which was a total of 6 matches in a 1-day tournament). All matches were 14 min (7 min halves). Championship finals were 10 min halves. Players received no remuneration for either training or playing.

### Injury Definitions and Data Collection Follow-ups

The methods were compliant with the consensus statement on injury definitions and data collection procedures for studies of injuries in Rugby [22]. To support inter-study comparison of injury types, a senior medical/graduate student and a resident-in-training coded injuries using the international sports coding system OSICS-10.1 [23].

Injuries were defined as “any physical complaint caused by transfer of energy that exceeded the body’s ability to maintain its structural and/or functional integrity, sustained by a player during a rugby match” [22]. Tournament injuries were classified as medical attention or time loss, with recurrent injury and time to return to sport defined as [22]:

- *Medical-attention injury:* a player received medical attention for an injury that resulted in no absence from play.
- *Time-loss injury:* an injury that results in a player being unable to take a full part in future rugby training or match play.
- *Injury severity:* the number of days (d) that elapsed from the date of injury to the date of the player’s return to full participation in team training and the player’s availability for match selection.
- *Recurrent injury:* an injury of the same type and at the same site as an index injury occurring after a player’s return to full participation from the index injury.
- *Time to return to sport:* when an injured player was medically cleared and returned to full-contact training or match play counted in days, as an indicator of injury severity.

All time-loss injuries were followed up at 1-, 3-, and 6-month intervals until they either returned to play or retired. Time to return to sport was counted in days from the day of injury [2, 11–13].

According to the Laws of Rugby, “A tackle occurs when the ball carrier is held by one or more opponents and is brought to the ground” [24]. Tackle types were considered “any event where one or more tacklers attempted to stop or impede the ball carrier whether or not the ball carrier was brought to ground,” and defined as [25]:

- *Arm tackle*: A tackler brings one arm across the chest of the ball carrier to prevent advancement.
- *Collision tackle*: A collision between tackler and ball carrier, while semi-erect, without tackler wrapping arms around ball carrier.
- *Jersey tackle*: A tackler only grabs the jersey of the ball carrier to prevent advancement.
- *Lift tackle*: A tackle where the tackler lifts the ball carrier’s hips above the level of their shoulders.
- *Shoulder tackle*: A tackler wraps his arms around the ball carrier’s body or lower extremity with head to the level and side of the ball carriers’ hip and drives through ball carrier.
- *Smother tackle*: A tackle by one or more players that wrap arms around ball carrier and prevents the ball carrier from passing or releasing the ball into play.
- *Tap tackle*: A tackler that taps the lower extremity (foot) of the ball carrier while running to make the ball carrier lose balance.

### Statistical Analysis

Data were de-identified, coded, and entered into a spreadsheet. Calculations and analyses were performed using Stata V.14.1 (Stata Inc., College Station, TX, USA). Injury exposure was calculated in playing hours (ph). Over 24 tournaments, a total of 643 matches were played, each lasting 14 min (0.23 h) in length. Overall, injury exposure for all tournament players was 2101 player-match-hours (ph) (7 players per side × 2 teams per match × 0.23 h per match × 643 matches) [22]. Prevalence was calculated by dividing injury by exposure and then multiplying by 100. Data incidence was reported as injuries per 1000 player-match-hours (1000 ph), percentages (%), means, standard deviations (SD), and confidence intervals (95% CI). Fisher’s exact tests compared incidence and prevalence ratios. Rate ratios compared injury incidences between subgroups. A single sample chi-square test was used to test the difference between observed and expected values for categorical variables. Fisher’s exact 2-sided tests assessed injury differences stratified by playing position. One-way ANOVA tests determined significance between means, and a Bonferroni post-hoc test provided pairwise comparisons. Significance for analyses was set at *p <* 0.05.

## Results

### Player Exposure

Match exposure was calculated from 3804 players on 317 teams (61 females, 256 males) who competed in 643 youth division matches (515 male; 140 female; 12 forfeits), over 24 Rugby-7s tournaments played in the USA (21 1-day and 3 three-day tournaments across 2010–2014). Match exposure of 2101 player-match-hours (ph) (1200 backs; 900 forwards) incorporated 1646 ph in males (backs 941 ph, forwards 706 ph) and 454 ph in females (backs 259 ph, forwards 194 ph).

### Player Characteristics

Rugby-7s injury match data were recorded for 39 females and 134 males (*n* = 173; mean 17.1 years; CI 16.9–17.3 years) (Table 1). Injured players found males were taller (*p <* 0.001), heavier (*p <* 0.001), and older (*p =* 0.034) than females, and forwards were heavier than backs for both sexes (male *p <* 0.001; female *p =* 0.021).

**Table 1:** Demographic characteristics (mean ± SD) for U.S. Rugby-7s U19 players with overall injuries combined as a function of sex and playing position

Sex and playing position	***N***	Age (years)	Stature (cm)	Body mass (kg)
**Female**	39	16.7 ± 1.2	165.5 ± 7.1	62.7 ± 10.3
Backs	16	16.4 ± 1.2	164.7 ± 6.4	58.2 ± 5.1
Forwards	15	17.1 ± 1.1	165.5 ± 6.8	67.6 ± 13.3
Missing^1^	8	16.7 ± 1.1	167.2 ± 9.8	62.8 ± 7.8
**Male**	134	17.2 ± 1.5	176.0 ± 8.0	78.8 ± 11.4
Backs	76	17.5 ± 1.3	175.0 ± 8.4	75.4 ± 10.7
Forwards	41	17.3 ± 1.4	177.5 ± 6.8	85.7 ± 10.2
Missing^1^	17	16.0 ± 1.6	176.5 ± 8.5	78.9 ± 10.6

^1^Players did not identify their playing position (backs or forwards)

### Overall, Medical-attention, and Time-Loss Injury Incidences

No fatal or catastrophic injuries were reported. Overall, injury incidence (time loss and medical attention combined) was 82.4/1000 ph (104 medical attention 49.5/1000 ph; 69 time loss 32.8/1000 ph; *p =* 0.008) (Tables 2 and 3). There was no difference in overall injury incidence between player position (backs 76.6/1000 ph; forwards 62.2/1000 ph; RR 1.2; CI 0.9–1.7; *p =* 0.218) or sex (males 81.4/1000 ph; females 85.9/1000 ph; RR 0.9; CI 0.7–1.4; *p =* 0.757). Medical-attention injury incidence was similarly found between player positions (backs 39.2/1000 ph; forwards 44.4/1000 ph; RR 0.9; CI 0.6–1.4; *p =* 0.557). Of 173 match injuries (39 females, 134 males), 69 (40%) were classified as time-loss injuries. Time-loss injuries by year were frequent, in 2010 (10.9/1000 ph), 2011 (27.8/1000 ph), 2012 (56.3/1000 ph), 2013 (153.0/1000 ph), and 2014 (8.3/1000 ph). Backs encountered more time-loss injuries (37.5/1000 ph) than forwards regardless of sex (17.8/1000 ph; RR 1.3; *p =* 0.008). No differences were found between player sex for time-loss (males 31.0/1000 ph; females 39.6/1000 ph; RR 0.8; CI 0.4–1.4; *p =* 0.369) or medical-attention injuries (males 50.4/1000 ph; females 46.2/1000 ph; RR 1.1, CI 0.7–1.9. *p =* 0.741). Time to return to sport lasting more than 1 week (i.e., an indicator of moderate or severe injury) accounted for 77% of all time-loss injuries (25.2/1000 ph; 45.0 days; CI 32.5–57.4). The mean time absent was 35.5 days, and whilst longer in males, was not significantly different between sexes (females 29.6 days; males 37.6 days; *p =* 0.505).

**Table 2:** Distributions for the location, type, and onset of medical-attention injury as a function of playing position by sex for U.S. Rugby-7s U19 players

Proportion; % (95% CI)						
	Males			Females		
Nature of injury	Backs (*n* = 39)	Forwards (*n* = 33)	All players^**1**^ (*n* = 83)	Backs (*n* = 8)	Forwards (*n* = 7)	All players^**1**^ (*n* = 21)
**Location**						
Head/neck	33.3 (19.9–50.1)	24.2 (12.1–42.6)	30.1 (21.1–41.0)	37.5 (8.7–79.2)	28.6 (4.2–78.5)	23.8 (9.5–48.3)
Upper extremity	28.2 (15.9–44.9)	30.3 (16.6–48.8)	28.9 (20.0–39.8)	12.5 (0.9–68.1)	14.3 (1.0–74.3)	28.6 (12.5–52.9)
Trunk	2.6 (0.3–17.4)	0 (–)	1.2 (0.2–8.4)	0 (–)	14.3 (1.0–74.3)	4.8 (0.6–30.9)
Lower extremity	33.3 (19.9–50.1)	42.4 (26.2–60.4)	37.3 (27.5–48.4)	37.5 (8.7–79.2)	42.9 (9.1–85)	38.1 (19.1–61.7)
Other	2.6 (0.3–17.4)	3.0 (0.4–20.3)	2.4 (0.6–9.4)	12.5 (0.9–68.1)	0 (–)	4.8 (0.6–30.9)
**Type**						
Bone	2.6 (0.3–17.4)	3.0 (0.4–20.3)	3.6 (1.1–10.8)	0 (–)	0 (–)	4.8 (0.6–30.9)
Joint (non-bone)/ligament	35.9 (22–52.6)	48.5 (31.4–65.9)	41.0 (30.7–52.0)	37.5 (8.7–79.2)	42.9 (9.1–85)	42.9 (22.6–65.8)
Muscle/tendon	28.2 (15.9–44.9)	27.3 (14.3–45.7)	25.3 (17.0–36.0)	12.5 (0.9–68.1)	14.3 (1.0–74.3)	19 (6.7–43.6)
Skin	23.1 (12.1–39.5)	12.1 (4.4–29.4)	18.1 (11.1–28.1)	12.5 (0.9–68.1)	28.6 (4.2–78.5)	14.3 (4.2–38.7)
Concussions	7.7 (2.4–22.2)	6.1 (1.4–22.6)	9.6 (4.8–18.3)	25.0 (4.1–72.4)	14.3 (1–74.3)	14.3 (4.2–38.7)
Other injuries	2.6 (0.3–17.4)	3.0 (0.4–20.3)	2.4 (0.6–9.4)	12.5 (0.9–68.1)	0 (–)	4.8 (0.6–30.9)
**Onset**						
Acute	92.3 (77.8–97.6)	100 (–)	95.2 (87.6–98.2)	100 (–)	85.7 (25.7–99.0)	90.5 (66.0–97.9)
Gradual	7.7 (2.4–22.2)	0 (–)	4.8 (1.8–12.4)	0 (–)	14.3 (1.0–74.3)	9.5 (2.1–34)

^1^Includes 11 male and 6 female players who did not specify a playing position

**Table 3:** Distributions for the location, type and onset of time-loss injury as a function of playing position by sex for U.S. Rugby-7s U19 players (*N* = 69; 51 males, 18 females)

Males						
	Proportion; % (95% CI)			Injury severity; mean days absent (95% CI)		
Nature of injury (***N*** = 51)	Backs (*n* = 37)	Forwards (*n* = 8)	All Players^**1**^ (*n* = 51)	Backs (*n* = 37)	Forwards (*n* = 8)	All Players^**1**^ (*n* = 51)
**Location**						
Head/neck	13.5 (5.5–29.4)	50 (17.9–82.1)	21.6 (12.1–35.4)	30.6 (0–81.6)	51.3 (0–137.4)	45.3 (16.6–73.9)
Upper extremity	32.4 (19–49.6)	12.5 (1.4–58.8)	31.4 (19.9–45.7)	51.3 (26.1–76.5)	36.0 (–)	46.9 (28.2–65.5)
Trunk	5.4 (1.3–20.2)	0 (–)	3.9 (0.9–15.0)	26.0 (26.0–26.0)	(–)	26.0 (26.0–26.0)
Lower extremity	48.6 (32.6–65)	37.5 (11.1–74.3)	41.2 (28.2–55.5)	31.8 (0–64.6)	14 (0–44.1)	29.2 (1.3–57.1)
Other	–	–	2.0 (0.3–13.4)	(–)	(–)	3.0 (–)
**Type**						
Bone	13.5 (5.5–29.4)	12.5 (1.4–58.8)	15.7 (7.9–28.9)	48.4 (25.3–71.5)	130.0 (–)	55.0 (26.4–83.6)
Joint (non-bone)/ligament	43.2 (27.9–60)	37.5 (11.1–74.3)	25.5 (15.1–39.6)	35.6 (0–72.6)	22.0 (0–64.4)	33.5 (2.8–64.2)
Muscle/tendon	27.0 (14.8–44.1)	12.5 (1.4–58.8)	2.0 (0.3–13.4)	40.7 (7–74.4)	12 (–)	40.7 (14.5–66.8)
Skin	2.7 (0.3–18.1)	0 (–)	2.0 (0.3–13.4)	21 (–)	(–)	21.0 (–)
Concussion	13.5 (5.5–29.4)	37.5 (11.1–74.3)	17.6 (9.2–31.1)	30.6 (0–81.6)	25 (0–65.0)	32.0 (7.4–56.6)
Other injuries	(–)	(–)	2.0 (0.3–13.4)	(–)	(–)	3.0 (–)
**Onset**						
Acute	100 (–)	100 (–)	100 (–)	37.6 (19.9–55.4)	35.4 (1.5–69.2)	37.6 (24.0–51.2)
**Females**						
	**Proportion; % (95% CI)**			**Injury severity; mean days absent (95% CI)**		
**Nature of injury (*N*** **= 18)**	**Backs** (*n* = 8)	**Forwards** (*n* = 8)	**All players**^**1**^ (*n* = 18)	**Backs** (*n* = 8)	**Forwards** (*n* = 8)	**All players**^**1**^ (*n* = 18)
**Location**						
Head/neck	37.5 (10.2–76)	25 (4.9-68.2)	27.8 (10.9–54.7)	41.3 (21.3–61.4)	51.0 (0.0–394.1)	45.2 (19.6–70.8)
Upper extremity	50 (16.6–83.4)	12.5 (1.2-62)	33.3 (14.4–59.7)	28.5 (0.0–62.4)	32.0 (–)	25.0 (4.5–45.5)
Trunk	0.0 (–)	0.0 (-)	0.0 (–)	0.0 (–)	0.0 (–)	0.0 (–)
Lower extremity	12.5 (1.2–62)	62.5 (24-89.8)	38.9 (18.2–64.5)	31.0 (–)	9.8 (0.0–21.3)	22.4 (0.0–46.9)
**Type**						
Bone	12.5 (1.2–62.0)	0.0 (-)	11.1 (2.4–38.9)	56.0 (–)	0.0 (–)	66.5 (0.0–199.9)
Joint (non-bone)/ligament	37.5 (10.2–76)	50 (16.6–83.4)	38.9 (18.2–64.5)	19.3 (0.0–52.4)	16.0 (0.0–38.8)	17.4 (5.6–29.3)
Muscle/tendon	12.5 (1.2–62)	37.5 (10.2–76)	27.8 (10.9–54.7)	31.0 (–)	31.7 (0.0–132.3)	26.7 (0.0–56.0)
Skin	0.0 (–)	0.0 (–)	0.0 (–)	0.0 (–)	0.0 (–)	0.0 (–)
Concussions	37.5 (10.2–76)	12.5 (1.2–62)	22.2 (7.7–49.5)	41.3 (21.3–61.4)	24.0 (–)	37.0 (19.7–54.3)
Other injuries	0.0 (–)	0.0 (–)	0.0 (–)	0.0 (–)	0.0 (–)	0.0 (–)
**Onset**						
Acute	100 (–)	100 (–)	100 (–)	33.6 (20.3–47.0)	22.9 (2.1–43.6)	29.6 (18.6–40.7)

^1^Includes 8 male and 2 female players who did not specify a playing position

### Injuries Defined as Acute, Gradual, New and Recurrent, and Removal from Play

Most overall injuries (time loss and medical attention combined) were acute (97%; 79.5/1000 ph) (Table 4). Of six (2.9/1000 ph) injuries that were overuse or gradual onset, none were classified as time loss. Most acute injuries (time loss and medical attention) seen among the U.S. Rugby U19 population were new injuries rather than a recurrent injury (new 77%, 63.8/1000 ph; recurrent 23%, 18.6/1000 ph; *p <* 0.001). Most recurrent injuries overall, of the same type and site (time loss and medical attention), were early recurrences (within 2 months of the original injury; overall 59%; females 57%; males 60%). When viewed in context of a given Rugby-7s season, recurrent injuries recurred most commonly from initial injuries during the players’ current rugby season (59%), previous rugby season (23%), another sport (15%), or outside of sports participation (3%). Following removal from the field, 18% of players required further care than on the field sidelines (urgent care setting 13%, admitted to hospital 3%, sent to physician 2%).

**Table 4:** Proportion and mean days absent before time to return to sport for injuries among U.S. Rugby-7 s U19 players as a function of match activity and playing position (N = 69, 45 backs, 16 forwards)

All players	Backs (*n* = 45)		Forwards (*n* = 16)		All players (*n* = 69)	
***Risk factor***	Proportion, % (95% CI)	Mean days absent (95% CI)	Proportion, % (95% CI)	Mean days absent (95% CI)	Proportion, % (95% CI)	Mean days absent (95% CI)
**Contact**	84.4 (70.1–92.6)	38.8 (22.1–55.5)	75 (45.7–91.4)	31.7 (7.7–55.6)	81.2 (69.9–88.9)	37.4 (25.2–49.7)
Impact with player	42.2 (28.3–57.5)	44.3 (13.8–74.9)	56.3 (29.8–79.6)	36.9 (4.9–68.9)	44.9 (33.4–57)	42.5 (22.5–62.6)
Impact with ground	8.9 (3.2–22.1)	19 (0–38.7)	12.5 (2.6–43)	5 (0–30.4)	10.1 (4.8–20.1)	18 (4.4–31.6)
Impact w/player and ground	33.3 (20.8–48.8)	37.1 (15.9–58.4)	6.3 (0.7–39.3)	38 (–)	26.1 (16.9–38)	36.3 (18.9–53.6)
**Non-contact**	6.7 (2.1–19.4)	13 (0–47.2)	6.3 (0.7–39.3)	3 (–)	5.8 (2.1–14.8)	10.5 (0–30.1)
Missing	8.9 (3.2–22.1)	37 (0–106)	18.8 (5.3–48.6)	27.7 (17.6-37.7)	13 (6.8–23.5)	34.6 (9.3–59.8)
**Phase of play**						
Maul	(–)	(–)	(–)	(–)	(–)	(–)
Ruck	(–)	(–)	12.5 (2.6–43)	57 (0–323.8)	4.3 (1.4–13)	39.3 (0–131.5)
Running/open play	8.9 (3.2–22.1)	33.3 (− 29.7–96.2)	25 (8.6–54.3)	21.5 (1.2–41.8)	11.6 (5.8–21.8)	27.4 (4–50.7)
Scrum	(–)	(–)	6.3 (0.7–39.3)	2 (–)	1.4 (0.2–10)	2 (–)
Tackle	86.7 (72.7–94.1)	37.6 (21.2–54)	56.3 (29.8–79.6)	29.3 (0–59.7)	76.8 (65.1–85.5)	37.2 (24.3–50)
Tackling	58.1 (42.5–72.3)	41.1 (15.6–66.6)	50 (21–79)	31.8 (5.5–58.2)	57.4 (44.4–69.4)	40.1 (21.6–58.6)
Tackled	41.9 (27.7–57.5)	34.6 (23.1–46.1)	50 (21–79)	36.5 (0–86.7)	42.6 (30.6–55.6)	34.6 (23–46.2)
Lineout	2.2 (0.3–15.1)	52 (–)	(–)	(–)	1.4 (0.2–10)	52 (–)
Missing	2.2 (0.3–15.1)	1(–)	(–)	(–)	4.3 (1.4–13)	30 (0–131.5)
**Removal from play**						
Immediate	40 (26.4–55.3)	33.6 (21.6–45.6)	56.3 (29.8–79.6)	25.2 (7.3–43.2)	47.8 (36.1–59.8)	33.9 (24.9–42.9)
Delayed	42.2 (28.3–57.5)	31.8 (13.9–49.8)	18.8 (5.3–48.6)	17.3 (0–60.2)	34.8 (24.3–47)	28.8 (14.4–43.2)
Not at all	(–)	(–)	6.3 (0.7–39.3)	24 (–)	1.4 (0.2–10)	24 (–)
Missing	17.8 (8.9–32.4)	56.5 (0–136.9)	18.8 (5.3–48.6)	54.3 (0–220.5)	15.9 (8.9–26.9)	55.9 (0–113.6)
**Injury onset**						
Acute	100 (–)	36.9 (22.4–51.5)	100 (–)	29.1 (11.5–46.7)	100 (-)	35.5 (25.1–45.9)

We did not analyze the eight players who did not answer the question regarding their playing position.

### Match Activities Involved with Injury

Most overall injuries (time loss and medical attention) were contact (78.0%; 64.3/1000 ph) versus non-contact (8.1%; 6.7/1000 ph; *p <* 0.001) (Table 4). Most overall injuries were a result of impact with another player (60%) as opposed to impact with some other entity (i.e., ground or ground and player) (40%; RR 1.5; *p* = 0.020). Time-loss injuries were more likely to occur due to injured player’s contact with another player, ground, or both player and ground compared to non-contact mechanisms (*p <* 0.001). No differences were found by injury severity and contact type (ground 18 days, player 42 days, or both 36 days; *p =* 0.445). In terms of match phase of play, tackling (76.8%) was the most injurious (tackling players 57.4%; tackled players 42.6%). Lineout injuries (52 days) had the longest time to return to sport. Jersey tackles (161 days) produced a longer time to return to sport than above the shoulder (18 days, *p =* 0.003), collisions (34 days, *p =* 0.003), and shoulder tackles (35 days, *p =* 0.002).

### Injury Types and Body Regions

The distribution of location, type, and onset of medical-attention injuries (Table 2) and time loss (Table 3) as a function of playing position for each sex are presented. Distribution of overall injuries (medical attention and time loss) based on body region injured found the lower extremity as the most common injured body region (39%), followed by upper extremity (30%), head and neck (27%), trunk (2%), and other (2%) (“Other” non-musculoskeletal match-encountered illnesses, e.g., exercise-induced asthma and heat illnesses). The most injured body parts overall (medical attention and time loss) were head/face (25%), ankle (15%), shoulder/clavicle (14%), and knee (12%). The most common overall (medical attention and time loss) injury types were ligament sprains (29%), concussions (14%), and hematomas/contusions/bruises (13%). The most common time-loss injuries were concussions (6.2/1000 ph; 34 days), ankle ligaments (5.7/1000 ph; 16 days), and shoulder ligaments (2.4/1000 ph; 23 days). Recurrent time-loss injuries (an injury of the same type and at the same site as an index injury) involved mostly joints and ligaments combined (41%), with sprained ligaments (29%, 2.4/1000 ph; 30 days) occurring more common than muscle strains (12%, 1.0/1000 ph; 16 days).

In terms of notable sex differences in injuries, a greater proportion of time-loss joint/ligamentous (male 25.5%, female 38.9%) and muscle injuries (male 2%, female 27.8%) were seen among female U19 Rugby-7 players compared to males. We otherwise did not find significant sex differences in terms of injured body system, part, or type of injury among the U19 population.

### Match Half and Multiple Match Tournament Day

Players overall time loss and medical attention sustained 90% of all injuries and 88% of all severe injuries (time loss > 28 days) during the first three matches of a tournament day (match 1, 29%; match 2, 26%; match 3, 34%).

## Discussion

Rugby’s popularity has expanded globally. The two popular formats of Rugby Union in the USA include Rugby-15s and Rugby-7s. Comparatively, there is more injury data on Rugby-15s among youths [17, 18, 20, 26]. There is currently no injury information on Rugby-7s and the U19 population. This is the first paper to provide an epidemiological profile of Rugby-7s U19 injuries and return to sport. As there is no injury profile for Rugby-7s adolescent players in the literature, comparisons with U.S. Rugby-7s community players were made to highlight potential differences between U.S. cohorts and U19 Rugby-7s participants for discussion purposes. Comparison to equivalent-aged Rugby-15s adolescent player’s injury incidence profiles were also made to draw distinction in injury pattern between the two Rugby formats for discussion purposes.

### Injury Incidences

Time-loss injury incidences of U.S. Rugby-7s U19 players were lower when compared to prior U.S. cohorts of community U.S. Rugby-7s (49.2–55.4/1000 ph) [2, 11, 13]. Time-loss (32.8/1000 ph) and medical-attention injury incidences (49.5/1000 ph) of U.S. Rugby-7s U19 were similar to those reported in literature for Rugby-15s: English boys (16–18 years old, 35–44/1000 ph), Under-18 players (U18) (42.86/1000 ph), and Under-20 (U20) players (49.7–57.2/1000 ph) [20, 27–29]. Comparing severity of match injuries (time to return to sport), incidence of severe injuries (moderate or severe, 21 to 28 days), among U.S. Rugby-7s U19 players was greater (25.2/1000 ph) than for New Zealand Rugby-15s adolescent players (1.1/1000 ph) [19] and had a longer time absent from play than Rugby-15s U18 players (20.8 mean days) [29]. U.S. U19 Rugby-7s players were therefore injured at similar rates to similarly aged cohorts [20, 27–29], but may have more severe injuries when a time-loss injury occurred. Caution however should be exercised in interpretation of severity data between playing cohorts as differences in playing seasons, schedules, and medical management in different countries or leagues may impact the data reported.

### Age and Size

The current U.S. U19 cohort’s mean age was similar to Rugby-15s adolescents in the literature [17]. The current study’s U19 cohort forwards and backs were shorter and weighed less than players in prior cohorts of international adolescent Rugby-15s [17, 20], but were comparable to an international U18 Rugby-15s playing cohort [29]. Differences in anthropometrics between the current U.S. U19 cohort and international players may reflect the different physical demands of the two codes, but may also be due to the existence of more structured training and conditioning programs for Rugby-15s in established countries, as compared to Rugby-7s in emerging rugby nations, which likely lack ancillary training for the Rugby-7s format. Many international training programs have position focused training, which would allow conditioning based on positional demands in the sport and its formats [30]. Established international rugby nations may also have a more structured training program for its youth, and therefore, able to provide more resources for their U19 cohorts, with structured contact-based training for positional demands, especially within set plays (including scrummaging and line-outs) and tackling techniques [30].

While anthropometric differences between study populations are readily reported, effects of these differences on population’s injury rates and risks are difficult to quantify. There is a perception that youth Rugby may be susceptible to mismatch in body composition and/or skill level, which may result in higher risks [5]. One possibility to mitigate injury risk due to the mismatch in body compositions in youth rugby would be to implement competition groups not based on age, but body composition and skill level [31]. Unfortunately, literature is limited in this area. Evidence is still evolving to establish efficacy of body mass-based criteria or playing down an age group in order to address the mismatch in junior Rugby [32]. Other injury preventive strategies in youth rugby include proposed rule changes to permit uncontested scrums in youth rugby, in order to avoid oversized packs of players against smaller sized packs [33].

### Body R**e**gion Injury Location and Type of Injuries

Rugby-7s, with its open format, requires repetitive bursts of sprinting, changes in direction along with acceleration and decelerations [2, 8]. Lower extremity injuries (39%) and ligament sprains (29%) were the most common injuries in the current cohort, which is consistent with literature on comparative aged U.S. high school and international Rugby-15s [17, 18]. These lower extremity rates were higher than a similar aged international U18 Rugby-15s (14%) [29] but lower than more competitive international U20 Rugby-15s players (47.3–50.6%) [27, 28]. Meanwhile, previous literature on U.S. community Rugby-7s noted lower rates (14.6%) [2] among lower extremity injuries than in elite Rugby-7s (47.1–56.3%) [6, 34] and Olympians (50–62.5%) [6].

A prior adult U.S. Rugby-7s cohort also encountered a high proportion of ligament injuries (25%) [2]. This reinforces the need for focused conditioning and warm-ups for our U19 cohort, which may reduce ligamentous injury in the demanding pace of the Rugby-7s format. In addition to positional differences in injuries, sex differences were also noted in the U.S. U19 Rugby-7s population. A higher proportion of time-loss joint/ligamentous (male 25.5%, female 38.9%) and muscle injuries (male 2%, female 27.8%) was seen among the female U19 Rugby-7 players, though, the differences were not significant. This likely reflects not only the need for better physical conditioning among the U.S. women’s population but also the adaption needed for female players to participate in a new collision sport. While U.S. men has U.S. Football, a popular female collision sport currently does not exist in the USA. This may be further compounded among U19 players with female participation [4], where there would be fewer programs for women, as compared to men, which may limit proper training and experience for female Rugby-7 players.

Comparing literature, the proportion of time-loss upper extremity injuries was higher in the current study (both sexes 31.9%, 41 days; males 31.4%, 47 days; females 33.3%, 25 days) than international populations, among adolescent boys Rugby-15s (academy 28.4%, 31 days; school 24.4% 52 days) and U18 Rugby-15s (14%; 9.33 days) [20, 29], but lower than competitive international U20 Rugby-15s players (27.1–28.2%) [27, 28]. Elite Rugby-7s players, National players and Olympians respectively, note upper extremity injuries to range between 17.8–28.8% and 25–35.7% [6, 34]. The U19 cohort was similar to U.S. Rugby-7s community player injury incidences of upper extremities (23.4–33.3%), which could reflect the amateur U.S. populations’ willingness to tackle with preconceived tackling views of other U.S. sports [2, 11]. Furthermore, U.S. U19 players may be newly introduced to the sport and the Rugby-7s format. This may reflect that U19 players are learning how to tackle, and skill sets may need to be nurtured in this population in a graduated or step-wise program [31]. Coaching may need to emphasize keeping elbows next to the trunk when tackling [35], and avoiding shoulder external abduction exposing the shoulder to potential glenohumeral injuries, reflective of poor tackling technique [36]. Due to player positions in rugby-15s, adolescent forwards were often injured in the tackle and direct impact situations, whereas backs were more often tackled at the point of trauma, with forwards more likely to suffer a recurrent injury [36]. Due to these facts, tackling technique is of utmost importance with skill developing players involved in the format of Rugby-7s.

In addition to upper extremity injuries, the proportion of time-loss head and neck injuries**,** including concussions, were also higher (combined 23.2%; males 21.6%; females 27.8%) among U.S. Rugby-7s U19 participants than U.S. Rugby-15s high school combined (both sexes 21.7%; males 22.1%; females 19.5%) [17], and international cohorts, male adolescent Rugby-15 (academy 13.8%, 13 days; school 17.8%) [20], U18 Rugby-15s (14%; 18 days) [29], and competitive U20 Rugby-15s (12.1–18.4%) [27, 28]. These injury proportions were lower than head and neck injury incidences reported in a previous U.S. community Rugby-7s cohort (31.3%) [2]. When considered in totality, both U.S. studies have reported higher proportions of head and neck injuries with Rugby-7s than reported numbers in international elite Rugby- s (4.9–23.2%) [6, 8, 34] as compared to Olympic players (12.5–14.3%) [6]. These findings may reflect limited training, developing tackling techniques, or lack of understanding of injury preventive biomechanics involved in U.S. Rugby-7 athletes of all ages, compared to international rugby players. Future interventions, including standardization of playing instruction to this age group, may help reduce head and neck injuries. U.S. rugby may have limited introductory instructional education on Rugby-7s, since many youth clubs may be player coached. Attempts to address this concern have been launched within the USA with standardized coaching course certifications on current rules and regulations [37].

Concussion injury rates in the U.S. U19 cohort were higher (males 17.6%, 32 days; females 22.2%, 37 days) than overall U.S. Rugby-7s concussion injury rates (14.6%) [2]. These were also higher than a comparative-aged U.S. High School Rugby-15s (combined 15.8%; males 16.1%; females 14.3%) [17], international U18 Rugby-15s (9.3%) [29], and U20 Rugby-15s (4.7-12.5%) players [27, 28]. These trends are a concern and need to be further evaluated with the literature documented high rates noted among student community rugby [26]. To note, Olympians in Rugby-7s encountering a concussion (7.1–12.5%) [6] were found at lower rates than U.S. Rugby-7s [2] and elite Rugby-7s (10.3–21.4%) [6]. Severity and time to return to sport after concussion for U.S. Rugby-7 s U19 players were similar among females (37.0 days; CI 19.7–54.3) and males (32.0 days; CI 7.4–56.6; *p* = 0.771) with a mean overall severity of 33.5 days (CI 17.3–49.7) for the entire cohort. The time to return to sport post-concussion in the U.S. U19 cohort was greater (males 32 days, females 37 days) when compared to international U18 Rugby-15s (17.5 days) [29]. Our prior U.S. community Rugby-7s cohort had a similar mean time to return to sport of 30.6 days post-concussion (men = 36.7 days; women = 27.9 days) [12]. International elite Rugby-7s has a wide range including 19.3–53 days absent in men’s Rugby-7s as compared to females (9 days) [8, 10, 34].

Recognition of concussions in U19 players, due to potential negative long-term effects, is a healthcare concern in adolescent rugby [26, 38]. Therefore, the fluctuating duration of time to return to sport among participation levels is possibly due to variability in suggested rest periods for ages and return to sport, post a mild traumatic brain injury. World Rugby’s concussion statement recommends variable times of rest by age, with one (1) week of rest minimally, among adults, meanwhile, two (2) weeks of rest among adolescents (i.e., 14 days seniors (U18), 14 days or longer for juniors (under-16) as a minimum), before beginning the gradual return to play (GRTP) program [39]. World Rugby suggests this could be addressed further by individual Rugby Unions or Nations where they may adjust these age levels upwards at their discretion [39]. Therefore, the New Zealand Rugby Union adds another week to this and states that World Rugby’s mandatory stand down period is for a minimum 3 weeks (23 days for players aged 19 and under) [40]. To aid in these concerns with concussions domestically, USA Rugby adheres to the World Rugby’s concussion statement [41].

Management of concussion at various organization levels (community/amateur versus professional) and countries likely varies and has evolved over time. This would impact the reported length of time to return to sport reported in various studies. These differences should be noted as it makes comparison of concussion severity across different player cohorts that exist at different times or at various competition levels likely difficult to interpret.

### Causes of Injury, Concerns with Tackling, and Recurrent Injury

Tackles during rugby are well-known risk factors for injury in Rugby-7s [7, 8]. The majority of injuries (78%) among the current U19 U.S. cohort occurred with tackling. Contact injuries in our population were more likely to result in time loss. While tackling resulting in majority of injuries are expected with rugby, the high number of collision-tackling injuries (players colliding with another player without attempting to wrap up the other player) in our U19 current population (17.2%) may reflect poor tackling techniques among the U.S. U19 players. Interestingly, a similar trend was seen among U.S. Rugby-7s adult-senior players (19.4%) [2]. Internationally, youth rugby typically starts contact at 7–9 years of age [5]. A taught approach to player contact/impact may be injury preventive [5]. Therefore, an emphasis on technical training in contact should be employed to the players newly introduced to the sport or re-emphasized among learning playing populations [42] to mitigate injuries, including high head injuries found among student age play [26]. To mitigate these injuries, a graduated step-wise program of developing contact techniques is needed to allow players to progress to higher levels in a safe manner between juniors and seniors [31].

The majority of match injuries encountered during U.S. U19 tournaments occurred during the first three matches of the tournament. The current study’s early match injury incidences may be explained by having less skilled and unconditioned teams being injured in early matches. Similar patterns of increased frequency of early match injuries have been reported in U.S. Rugby-7s amateur players [2, 11]. The majority were acute new injuries, consistent with literature on U.S. Rugby-7s amateur players and Rugby-15s adolescent players [2, 17].

Recurrent injuries, which were injuries of the same type and at the same site as an index injury, accounted for 20% of overall injuries in our U19 population [22]. Most recurrent injuries (59%) were re-aggravated within 2 months of a previous injury and during the same rugby season. Recurrent injury incidences were found at overall (including medical attention) 18.6/1000 ph (overall females 15.4/1000 ph; overall males 19.4/1000 ph; RR 1.3; CI 0.5–3.4; *p* = 0.603) or time loss 8.1/1000 ph (females 6.6/1000 ph; males 8.5/1000 ph; RR 1.3; CI 0.4–7.0; *p* = 0.737). There are no comparable youth populations in Rugby-7s in the literature; however, our youth Rugby-7s recurrent injury rates are both higher than international Rugby-15s boys’ academy (5/1000 ph) and school (4/1000 ph) cohorts [20]. The proportion of recurrent injuries at 59% from the current rugby season in our current youth cohort emphasizes the importance of the injured U.S. U19 players completing a full-length rehabilitation program prior to return to sport. U.S. youth rugby is currently mainly a club sport and involves traveling to venues. Hence, medical care post-tournament for teams traveling to tournaments is often lacking or difficult to coordinate after the player returns to their home region. An area of future potential influence, amongst this age, would be at the high school administration level, where in the USA, a majority of high schools have athletic trainer’s on-staff. This is ideal for adolescent rugby programs as it would provide more resources for education, intervention, and emphasis on adherence to current return to sport regulations.

### Concerns with U.S. Expansion Without an Injury Evidence-Based Approach

Exponential growth in U.S. rugby participation and exposure from 2010 (88,000 members) to 2016 (1.7 Million members) [1, 3] has resulted in an increasing number of events and fixtures. These circumstances expose players to a sport injury risk, which unlike other mainstream U.S. sports is largely not defined for rugby in the USA. This undefined risk may be a public health concern especially when one considers the public perception and landscape around head injuries in contact sports [43, 44]. Overall, safety and current changes in laws of the game have not been evaluated in emerging rugby-playing nations amongst players not accustomed to the sport. The current study on U.S. U19 players has shown that the U19 cohort has a similar injury rate compared to U.S. adult Rugby-7 participants. Both cohorts have high proportions of head and neck injuries. Proportions of ligamentous and muscle injuries among the U19 participants were also high in Rugby-7s. Continued exponential growth of Rugby-7s without understanding the evolving risk from participation exposure to the playing population would potentially result in a missed opportunity to intervene in injury prevention for this cohort.

This study supports implementation of community-based rugby injury surveillance. This directly refutes the view that community studies are limited, and rugby injuries cannot be documented due to rugby being an amateur sport that relies on mostly volunteer officials. A major concern is the lack of a detailed network or structure for injury surveillance. A detailed research-driven study with data would aid in the collaborative formulation of recommendations for improving the safety of adolescent players. This could be disseminated among the rugby-playing community for translation of injury preventive measures. Prior to earmarking further resources toward playing expansion, there should be a focused approach to understanding injury incidences, mechanisms, and risks involved in Rugby-7s.

### Limitations of this Study

A large study that utilizes multiple healthcare providers, despite being trained and provided with a detailed study manual, may be subject to inter-rater reliability concerns. This could affect definition interpretations and possibly influence injury incidences. Underreporting from unsupervised research data collectors at events may influence injury incidences. A break in the translation of study methodology and significance, to parents, players, and coaching staff, may also induce underreporting by all groups. Long-term studies on this U19 cohort will be needed and may provide future findings that evolve over time, to help understand injury preventive trends between sex, on this expanding playing cohort. Lack of training exposure data limits knowledge in this area. A potential perception that outcomes of the current study would hinder the sport’s growth and stagnate recruitment and retention of players which may also affect compliance by officers, staff, and players, with reporting injuries. Knowledge and support by the local governing organizations are needed to convey to the community the importance of injury surveillance work and implications of findings for safe growth of rugby in America.

### Summary of Key Points

A high prevalence of adolescent head/neck injuries, including concussions, was found for both males and females in our U19 cohort. These adolescent head/neck injury rates support the need for further investigation and intervention to reduce injuries among adolescents in U.S. Rugby-7s. More knowledge must be obtained on injurious tackle techniques and contact in general between players, among this young learning population. Proper tackling instruction is important in adolescent rugby players. U.S. Rugby-7s U19 would benefit from similar public health initiatives that exist to reduce head/neck injuries in other popular sports.

The data indicates that proper post-tournament management of youth Rugby-7s participants is important. More awareness and understanding needs to be conveyed to players and staff, as well as application of return to sport policies, to help decrease injury incidence, recurrent injuries, and therefore, severity. Players and coaching staff must be made aware of the conditioning needed to perform in this format, which would possibly mitigate early tournament injury rates and severity. Ligament injury incidences between current cohorts highlight the importance of population and sex-specific injury research programs in order to identify specific trends and optimize injury prevention efforts.

## Conclusions

This study is the first to report injury incidence for Rugby-7s U19 players. The overall match injury incidence among U.S. U19 Rugby-7s tournaments was similar compared to adult U.S. community Rugby-7s. The most common time-loss injuries seen among U19 tournaments were concussions (6.2/1000 ph; 34 days), ankle ligaments injuries (5.7/1000 ph; 16 days), and shoulder ligaments injuries (2.4/1000 ph; 23 days). Recurrent injury risk overall was 23% in this adolescent population. There were no significant differences in injury incidences between males and females. The first three matches of a tournament day result in the most injuries. Knowledge of these injury patterns in U19 Rugby-7s will help identify areas to direct resources to enable growth of Rugby-7s in youths and emerging countries being exposed to Rugby-7s. Community injury surveillance studies are essential to understand risks from participation in amateur sports.

## Data Availability

An anonymized summary of the datasets used and/or analyzed during this study may be available from the corresponding author on reasonable request. Please contact the authors for data requests.

## Abbreviations

U19: Less than 19 year-old tournament players
1000 ph: Injuries per 1000 player-match-hours
D(d): Days absent from play
RISE: Rugby Injury Survey and Evaluation report
RC: Research coordinator
RDC: Research data collector
GU: USA Rugby Geographic Union
TU: USA Rugby Territorial Union
CR: USA Rugby Competitive Region
